# The Transcriptional Regulator Rok Binds A+T-Rich DNA and Is Involved in Repression of a Mobile Genetic Element in *Bacillus subtilis*

**DOI:** 10.1371/journal.pgen.1001207

**Published:** 2010-11-11

**Authors:** Wiep Klaas Smits, Alan D. Grossman

**Affiliations:** Department of Biology, Massachusetts Institute of Technology, Cambridge, Massachusetts, United States of America; Universidad de Sevilla, Spain

## Abstract

The *rok* gene of *Bacillus subtilis* was identified as a negative regulator of competence development. It also controls expression of several genes not related to competence. We found that Rok binds to extended regions of the *B. subtilis* genome. These regions are characterized by a high A+T content and are known or believed to have been acquired by horizontal gene transfer. Some of the Rok binding regions are in known mobile genetic elements. A deletion of *rok* resulted in higher excision of one such element, *ICEBs1*, a conjugative transposon found integrated in the *B. subtilis* genome. When expressed in the Gram negative *E. coli*, Rok also associated with A+T-rich DNA and a conserved C-terminal region of Rok contributed to this association. Together with previous work, our findings indicate that Rok is a nucleoid associated protein that serves to help repress expression of A+T-rich genes, many of which appear to have been acquired by horizontal gene transfer. In these ways, Rok appears to be functionally analogous to H-NS, a nucleoid associated protein found in Gram negative bacteria and Lsr2 of high G+C *Mycobacteria*.

## Author Summary

There are several mechanisms by which bacteria acquire exogenous DNA. Sometimes this genetic material is advantageous for bacterial cells, for example, by making them resistant to antibiotics. Other times, foreign DNA has genes that are deleterious to the new host. Bacteria have mechanisms for helping to silence exogenously (horizontally) acquired genes. Many horizontally acquired genes are A+T-rich, a feature which can be important in distinguishing these loci from the host genes. We found that the transcriptional regulator Rok in the bacterium *Bacillus subtilis* preferentially binds to A+T-rich DNA. Together with previous work, our findings indicate that Rok helps repress expression of A+T-rich genes, many of which are likely to have been acquired by horizontal gene transfer. In these ways, Rok appears to be a functional analogue of the H-NS protein found in Gram negative bacteria (e.g., *E. coli*) and Lsr2 found in the high G+C *Mycobacterium tuberculosis*.

## Introduction

In bacteria, horizontal gene transfer typically occurs through natural transformation, conjugation, and transduction and contributes to rapid evolution and the acquisition of new traits [1], [2]. For example, genes needed for pathogenesis, symbiosis, resistance to antibiotics, and metabolism of various compounds are often acquired through horizontal gene transfer. Even though horizontal gene transfer can confer potential benefits on a recipient cell, there are potentially lethal costs. For example, the acquisition and expression of genes for restriction enzymes (often carried on phage) is potentially lethal. The acquisition of transcriptional regulators could lead to inappropriate rerouting of gene regulatory networks, and the introduction and expression of enzymes could lead to altered metabolic flux, resulting in loss of fitness under some conditions. In addition, the introduction and expression of homologues of essential genes may be detrimental to the cell if the gene products interfere with essential cellular components.

Many organisms have mechanisms to inhibit acquisition and expression of foreign DNA. These mechanisms can help to mitigate some of the potentially deleterious effects of acquisition and expression of foreign genes. For example, the H-NS protein of Gram-negative bacteria is a widely studied nucleoid-associated protein that modulates gene expression and is a negative regulator of some genes and mobile elements acquired by horizontal gene transfer [3]–[5]. The presence of H-NS appears to be confined to proteobacteria. A functional analogue has been recently described from the high-G+C Gram positive actinomycete *Mycobacterium tuberculosis* [6], [7], but no analogous proteins have been described in low-G+C Gram positive organisms to date.

We found that the transcription factor Rok of the low-G+C (43.5%) Gram positive *Bacillus subtilis* binds to A+T-rich DNA and helps repress the activity of at least one mobile genetic element. Rok is relatively small (20.7 kD) and is found in several Bacillus species closely related to *B. subtilis* [8]. Rok was previously identified as a negative regulator of natural genetic competence in *B. subtilis* [9]. It binds to and represses the promoter of *comK* [9]. *comK* encodes a transcriptional activator that is required for expression of the *B. subtilis* competence machinery needed for uptake of exogenous native and foreign DNA [10], [11]. In addition to repressing transcription of *comK*, Rok represses transcription of several other genes, including many that encode extracellular functions [8]. Rok binds specifically to the promoters of at least a subset of these genes, although a consensus recognition sequence has not been identified [8].

To better understand the function of Rok in gene regulation and cell physiology, we characterized the association of Rok with chromosomal DNA in vivo. We expected to find Rok binding largely limited to chromosomal regions of genes known to be transcriptionally regulated by Rok [8]. In addition, we found extensive binding of Rok to genomic regions characterized by a high A+T, many of which are believed to have been acquired via horizontal gene transfer. We also found that Rok binds to and represses endogenous excision of the mobile genetic element ICE*Bs1*, an integrative and conjugative element (conjugative transposon) found integrated in the 3′-end of a tRNA gene in *B. subtilis* [12], [13]. Our results indicate that Rok is a nucleoid-associated protein, is bound to chromosomal regions containing A+T-rich sequences, and inhibits the activity of at least one mobile genetic element. In these ways, Rok helps to repress several genes and elements known or thought to have been acquired by horizontal gene transfer.

## Results

### Rok is associated with parts of the nucleoid

**Figure 1:**
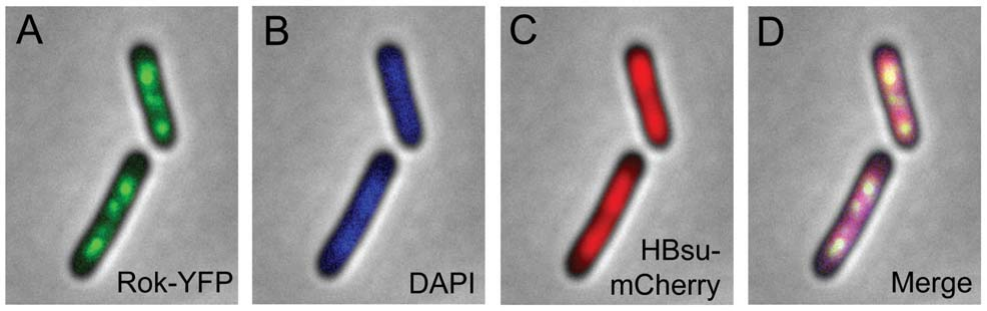
Localization of fluorescent Rok and HBsu fusion proteins. Strain WKS1102 (*rok-iyfp*; *hbs-mcherry*) was grown to mid-exponential phase at 32°C in defined minimal medium with glucose. Fluorescence microscopy images are overlaid with the phase contrast image. Images are representative of >95% of the cells. A. Rok-IYFP. B. DAPI. C. HBsu-mCherry. D. Merged image of A–C.

Rok is a DNA binding protein and appears to have some sequence specificity, although no consensus binding sequence has been identified [8]. We used fluorescence microscopy to visualize the location of Rok in living cells (Figure 1A). We fused *rok* to *yfp*, (encoding yellow fluorescent protein, IYFP [14] such that *rok-yfp* was expressed from its native promoter and was the only source of Rok in the cell. Strains expressing the fusion had normal transformation frequencies, indicating that Rok-YFP was functional (data not shown). As expected for a DNA-binding protein, Rok-YFP (Figure 1A) appeared to be associated with the nucleoid, as visualized with 4′,6-diamidino-2-phenylindole (DAPI) (Figure 1B). However, Rok-YFP was not uniformly associated with the nucleoid (Figure 1).

In contrast, the nucleoid binding protein HBsu, the *B. subtilis* homologue of HU from Gram negative organisms [15], appeared to be associated with the nucleoid in a relatively uniform manner (Figure 1C, 1D). We fused HBsu (*hbs*) to mCherry [16] such that *hbs-mcherry* was expressed from the endogenous *hbs* promoter and the fusion was the only source of HBsu in the cell. *hbs* is essential for cell growth [17], and the *hbs-mcherry* fusion strain appeared to grow normally, indicating that the fusion was functional. Since HBsu-mCherry appeared to be associated with most or all of the nucleoid, and Rok-YFP was not uniformly associated with the nucleoid (Figure 1), we inferred that the DNA binding preferences of these two proteins are likely to be different. This was further explored in chromatin immunoprecipitation experiments with HBsu and Rok described below.

### Rok associates with A+T-rich chromosomal DNA

**Figure 2:**
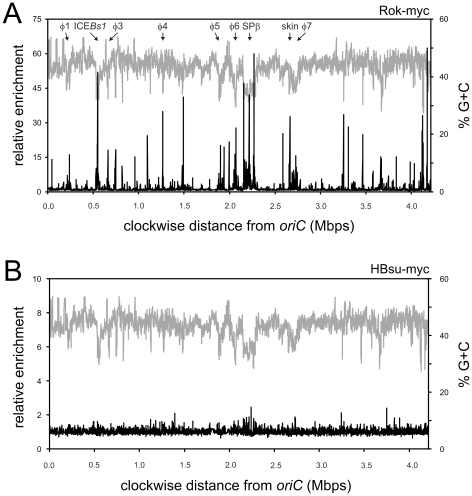
Genome-wide binding profile of Rok and Hbs. Strains WKS895 (*rok-myc*) and WKS935 (*hbs-myc*) were grown to mid-exponential (OD_600_∼0.6) phase at 32°C in defined minimal medium with glucose and processed as described in Materials and Methods. Relative enrichment of a particular genomic region is shown on the left axis, percent G+C on the right axis, and genome position on the x-axis. Enrichment values are averages from three independent cultures. A. Rok-myc (black curve) in relation to a sliding window analysis of the % G+C of the *B. subtilis* genome (gray curve). B. ChIP-chip of HBsu-myc (black curve) in relation to a sliding window analysis of the % G+C of the *B. subtilis* genome (gray curve).

We determined the genome wide binding profile of Rok (Figure 2A) and HBsu (Figure 2B) in vivo using formaldehyde-mediated crosslinking and immunoprecipitation (ChIP) and hybridization of immunoprecipitated DNA to DNA microarrays (ChIP-chip). We fused a cMyc epitope tag to the 3′ end of *rok* (*rok-myc*) and *hbs* (*hbs-myc*) such that each fusion is expressed from its native promoter and is the only copy of the gene in the cell. Like Rok-YFP and HBsu-mcherry, both myc-tagged proteins appeared to be functional; *rok-myc* strains had normal levels of competence and *hbs-myc* strains were viable (data not shown). We used monoclonal anti-myc antibodies to immunoprecipitate the myc-tagged proteins and detected DNA that was co-precipitated using DNA microarrays containing >99% of the annotated *B. subtilis* open reading frames as well as a subset of intergenic regions [18].

We found that Rok was associated with several genomic regions (Figure 2A), many of which correlated with the locations of genetic elements known or thought to have been acquired by horizontal transfer [19], [20]. Since horizontally acquired sequences often have a different G+C content than endogenous genes, we compared the Rok binding profile to the G+C content of the genome (Figure 2A). We calculated the average G+C content in windows of 3,000 bp with a step-size of 1,000 bp (Materials and Methods). The average % G+C across the entire genome is ∼43.5% [19]. Many of the chromosomal regions associated with Rok were strikingly lower in % G+C (higher % A+T) than the rest of the genome (Figure 2A).

The observed binding profile was specific for Rok, and not the myc-epitope tag or nucleoid binding proteins in general as the binding profile of HBsu-myc (Figure 2B) was quite different from that of Rok-myc (Figure 2A). In contrast to Rok, none of the chromosomal regions had >2.5-fold enrichment for HBsu-myc (Figure 2B), consistent with the function of HBsu as a general nucleoid binding protein [21]. Our results indicate that in vivo, HBsu is a relatively non-specific DNA binding protein and Rok binds preferentially to A+T-rich regions of the chromosome.

**Figure 3:**
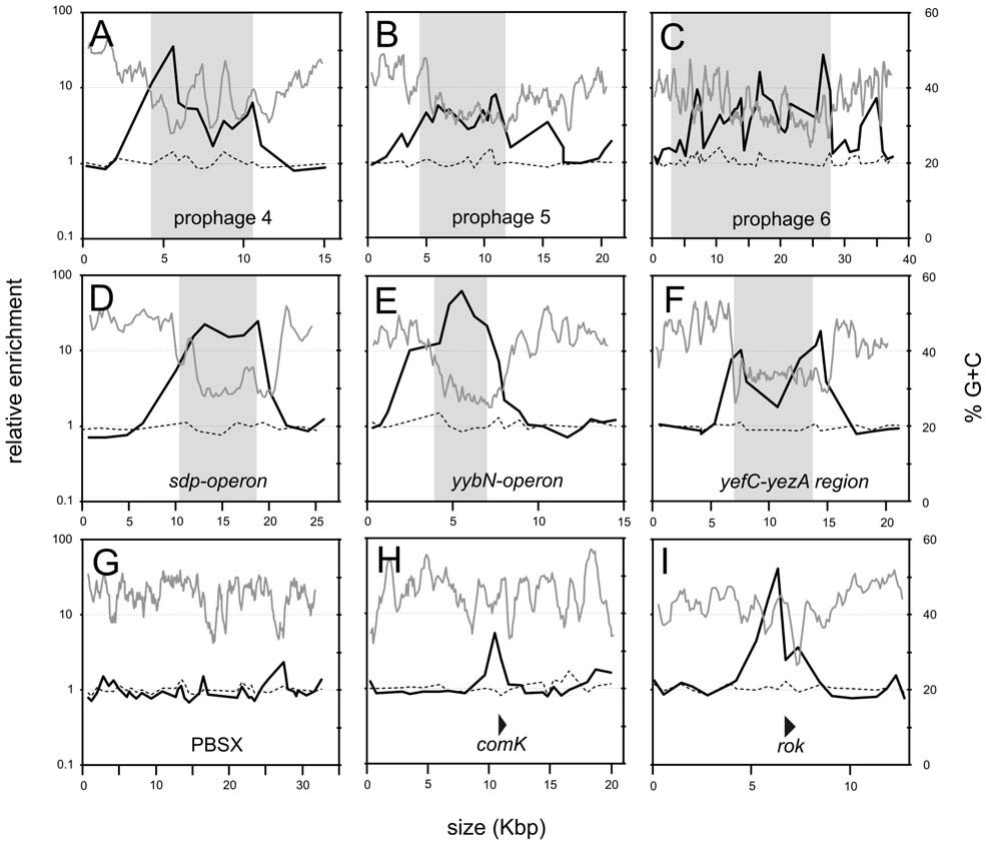
Detailed view of selected chromosomal regions. ChIP-chip data from Figure 2 are shown in more detail for selected chromosomal regions. The length of the genomic region is indicated on the x-axis. Enrichment in a Rok-myc immunoprecipitate (black curve) and HBsu-myc immunoprecipitate (dashed line) are given in relation to a sliding window analysis (window of 500 bp and a step size of 100 bp) of the % G+C of the *B. subtilis* genome (gray curve). A–F. Regions known or proposed to have been acquired by horizontal transfer [20] are indicated with a shaded box. A. Prophage 4 region (*yjcJ-manA*). B. Prophage 5 region (*ynbA-yncB*). C. Prophage 6 region (*yoaW-yobS*). D. *sdp* region (*opuBD-opuCA*). E. *yybN* region (*yybP-yybA*). F. *yefC*-*yezA* region (*yeeA-yeeK*). G-I. PBSX, a defective prophage, *rok*, thought to have been acquired by horizontal gene transfer, and *comK*, required for development of genetic competence. The locations and direction of transcription of *comK* and *rok* are indicated by triangles. G. PBSX region (*yjqA-ykaA*). H. *comK* region (*yhfO-yhjJ*). I. *rok* region (*ykuN-yknU*).

Analysis of the ChIP data for smaller chromosomal intervals highlights the difference between Rok and HBsu binding (Figure 3). Some of the chromosomal regions have genes characteristic of prophages, or defective prophages and thus appear to be or once have been functional mobile genetic elements likely acquired by horizontal gene transfer. These regions were designated as prophage regions and given a number [19]. Prophage regions 4, 5, and 6 have regions of G+C content significantly less than 40% (using a sliding window of 500 bp and a step size of 100 bp) and these regions are preferentially bound by Rok, but not HBsu (Figure 3A–3C). Similarly, the defective prophage skin [22]) and the prophage regions 1 and 7 also preferentially bind Rok, but not HBsu (Figure 2 and data not shown). Regions of the prophage SPβ [23] were bound by both Rok and HBsu, although binding of Rok appeared to be much greater (Figure 2). The *sdp* operon (Figure 3D), *yybN* operon (Figure 3E), and the *yefC-yezA* region (Figure 3F) are postulated to have been acquired by horizontal gene transfer [20]. Rok binds these regions in vivo, and binding correlates well with the regions of low G+C content (Figure 3D–3F).

Rok does not bind to all horizontally acquired DNA. The defective prophage PBSX [24] is a mobile genetic element likely acquired by horizontal gene transfer and Rok was not significantly bound to this chromosomal region (Figure 3G). However, the nucleotide composition of the PBSX region is not significantly different from the average of the *B. subtilis* genome (Figure 3G), consistent with the findings that Rok binds preferentially to regions of relatively low G+C content. Similarly, H-NS in *Salmonella* species does not significantly associate with mobile genetic elements that have G+C content similar to that of the host [25], [26].

### Rok association with previously defined targets

Previously, Rok was found to control expression of at least 20 different transcription units, either directly or indirectly [8], [9]. At least nine of these appear to be directly regulated by Rok since Rok binds to the promoter regions in vitro [8]. The ChIP-chip data indicate that Rok is also bound to (or near) almost all of these in vivo (Table 1), consistent with a role for Rok in directly repressing their expression.

**Table 1:** Association of Rok in vivo with specific transcription units.

First gene of cluster	genes in putative transcription unit	>2-fold enriched
*comK*	*-*	*comK*
*rok*	*-*	*rok*
*sboA*	*sboX, albA, albB, albC, albD, albE, albF, albG*	*{sboA, sboX}, albA*
*bhlA*	*bhlB?*	*bhlA, bhlB*
*sunA*	*sunT, bdbA, yolJ, bdbB*	*{sunA}, sunT, bdbA, yolJ, bdbB*
*yydH*	*yydI, yydJ*	*yydH, yydI, yydJ*
*yybN or yybM*	*yybL, yybK*	*yybN, yybM, yybL, yybK*
*yxaJ*	*yxaL*	*yxaJ*
*ykuJ*	*ykuK, ykzF*	*
*sdpA (yvaW)*	*sdpB (yvaX), sdpC (yvaY)*	*sdpA, sdpB, sdpC*

The first gene of most of the putative transcription units that were previously found to have increased mRNA levels in *a rok* null mutant [8] are listed in the first column followed by other genes in the putative operons in the second column. Those genes that were enriched >2-fold in the ChIP-chip experiments (data from Figure 2A) are shown in the last column.

Brackets {} indicate genes that were absent from the data set. They are included because regions flanking them were associated with Rok.

*ykuJ was enriched 1.8-fold.

Several of the previously identified Rok targets are located in genomic regions that have extensive binding of Rok in vivo. For instance, prophage 4 contains *yjcN*, a gene previously identified as a direct target of Rok [8]. Rok binding extends well beyond a simple regulatory region for *yjcN* (Figure 3A). Similarly, the *sdp* operon and *yybN* genomic region were previously identifie*d* as direct targets of Rok [8]. Rok binding to these regions in vivo extends greater than 5 kb (Figure 3D and 3E).

Rok also appears to bind to some regions that do not have significantly lower G+C content than the average for the *B. subtilis* genome. For example, the *comK* region has normal G+C content and Rok binds to this region in vivo (Figure 3H) and in vitro [9]. This also seemed to be the case for Rok binding to its own promoter region, although there is also some Rok binding in an A+T-rich region of *rok* (Figure 3I). These results might reflect recruitment of Rok by other DNA binding proteins. Alternatively, they might indicate that Rok has some DNA sequence-specific binding beyond the recognition of low G+C content, and that binding specificity could be obscured by the less specific and widespread association with low G+C content DNA.

### Rok associates with A+T-rich DNA in a heterologous host

**Figure 4:**
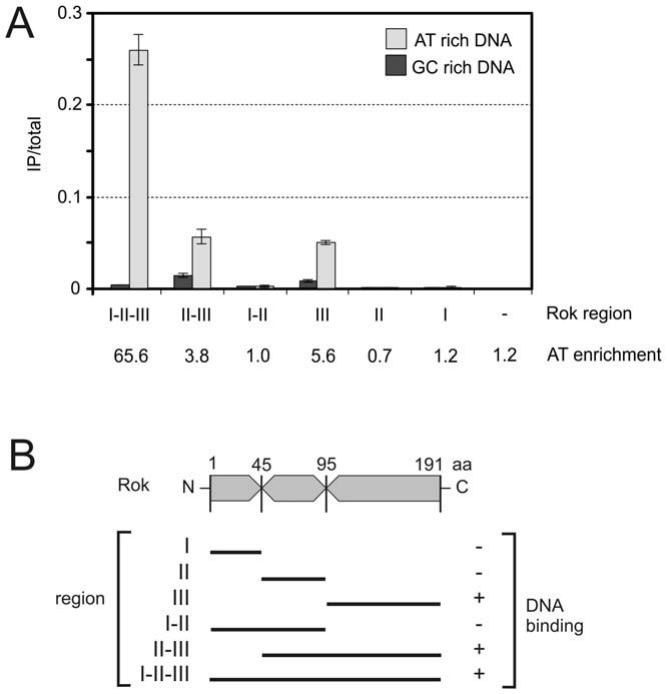
Preferential binding of Rok to A+T-rich DNA in *E. coli*. *E. coli* cells containing the indicated plasmids were grown to mid-exponential phase at 32°C in LB medium and processed for ChIP-PCR as described in Materials and Methods. A. Normalized levels of A+T rich (*ybcL*; 43.3% G+C; light gray) or G+C rich (*phnO*; 62.9%G+C; dark gray) DNA in the immunoprecipitates of the myc-tagged region I (pWKS1046), region II (pWKS1048), region III (pWKS1049), region I+II (pWKS1051), region II+III (pWKS1052) and full length Rok – region I+II+III (pWKS1054; same data as in Table 2). The relative enrichment of *ybcL* (A+T-rich) vs *phnO* (G+C-rich) is given below the graph. Error bars indicate standard error of the mean (n = 3). B. Schematic representation of the regions of Rok as defined for these experiments. DNA binding is indicated on the right.

We found that when expressed in *Escherichia coli*, Rok also bound preferentially to A+T-rich DNA. We introduced a plasmid expressing full length Rok with a C-terminal myc-tag (Rok-myc) into *E. coli* and confirmed by Western blotting that the protein accumulated (data not shown). We used ChIP followed by quantitative real time PCR (ChIP-qPCR) to compare the association of Rok-myc with six different chromosomal regions in *E. coli*, three that are A+T-rich and three that are G+C-rich (Table 2). There was significantly greater association of Rok-myc with the A+T-rich sequences than the G+C-rich sequences (Table 2; Figure 4A). This preferential binding of Rok-myc to A+T-rich sequences in *E. coli* is consistent with the binding of Rok to AT-rich sequences in *B. subtilis* as determined by ChIP-chip (Figure 2).

**Table 2:** Rok preferentially binds A+T-rich DNA in *E. coli*.

Locus (% A+T)^1^	% A+T (region)^2^	Signal in qPCR +/− S.E.^3^
*ybcL* 4 (56.7)	60.4 (5 kb)	0.260 +/− 0.017
*manC* (57.9)	65.1 (12 kb)	0.177 +/− 0.008
*rhsE* (52.0)	55.6 (4 kb)	0.204 +/− 0.002
*phnO* 4 (37.1)	40.3 (9 kb)	0.004 +/− <0.001
*yncH* (57.4)	42.5 (3 kb)	0.064 +/− 0.005
*ybbA* (39.9)	41.3 (5 kb)	0.035 +/− 0.005

1 The genetic locus in *E. coli* that was tested with PCR primers in quantitative real time PCR. The first three are in A+T-rich regions and the second three are in G+C-rich regions. Each primer set (Table S1) amplified a fragment between 101 bp–142 bp and the percent A+T of the amplified fragment is indicated.

2 The overall % A+T for a region of the indicated size surrounding the amplified fragment is shown. % A+T was determined using SWAAP (Materials and Methods).

3 The qPCR signal is indicated in arbitrary units based on a standard curve of total chromosomal DNA from the same strain. S.E.  =  standard error of the mean (n = 3).

4 Data for *ybcL* and *phnO* are the same as those presented in Figure 4A for full length Rok-myc.

### The C-terminal domain of Rok is sufficient for DNA binding

It is not known which region of Rok is needed for DNA binding [8], [9], [27]. Using various tools for sequence analyses combined with *rok* deletions and ChIP-qPCR, we found that the DNA binding activity of Rok is contained in the C-terminal region of the protein. We used a ClustalW2 alignment {http://www.ebi.ac.uk/clustalw/} of primary amino acid sequences of Rok homologs from *B. subtilis*, *B. amyloliquefaciens*, *B. pumilus*, *B. licheniformis*, *B. coagulans* and *B. pseudomycoides* to define conserved regions of the protein (Figure S1). The analysis revealed three distinct regions: (I) a highly conserved N-terminal region; (II) a moderately conserved central region; and (III) a highly conserved C-terminal region (Figure 4B and Figure S1). On the basis of the presence of many positively charged residues, we used the third putative domain to query the I-TASSER server for protein structure prediction [28]. The *in silico* analysis predicted that the C-terminal region might have some structural relatedness (Tm-score 0.6145) to winged helix DNA-binding domains of proteins such as FurB from *Mycobacterium tuberculosis* (PDB 2o03_A) {Lucarelli 2007, FurB}. The C-terminal region of Rok was also classified as containing a possible DNA/RNA-binding 3-helical bundle from the Winged Helix Superfamily, with an estimated precision of 20% by the PHYRE Protein Fold Recognition server [29].

Based on the in silico sequence analysis, we tested for the ability of the three regions of Rok to bind DNA. We fused each of these regions to a myc-epitope tag (Figure 4B) and used ChIP-qPCR to measure the ability of each fusion to associate with a region of *E. coli* DNA with high or low A+T content (as described above). Each of the fusion proteins accumulated to similar levels in *E. coli* and was detectable with anti-myc antibodies (data not shown). We found that any variant that contained the C-terminal region (region III) of Rok was able to bind DNA, and there was a preference for DNA with high A+T content (Figure 4A). In contrast, variants that contained region I, region II, or both, did not appear to have significant DNA binding activity (Figure 4A, 4B). However, association of full length Rok with A+T-rich DNA was greater than that of region III alone (Figure 4A). Based on these results, we conclude that region III of Rok is the DNA binding region and that this region alone has some preference for A+T-rich DNA. We suspect that region I, either alone or in combination with region II, likely contributes to DNA binding and the specificity for A+T-rich DNA, perhaps by affecting dimerization and/or multimerization of Rok and potentially contributing to cooperativity. This would be similar to H-NS and H-NS-like proteins where the N-terminal region affects multimerization [30].

### Rok contributes to stability of ICE*Bs1*

**Figure 5:**
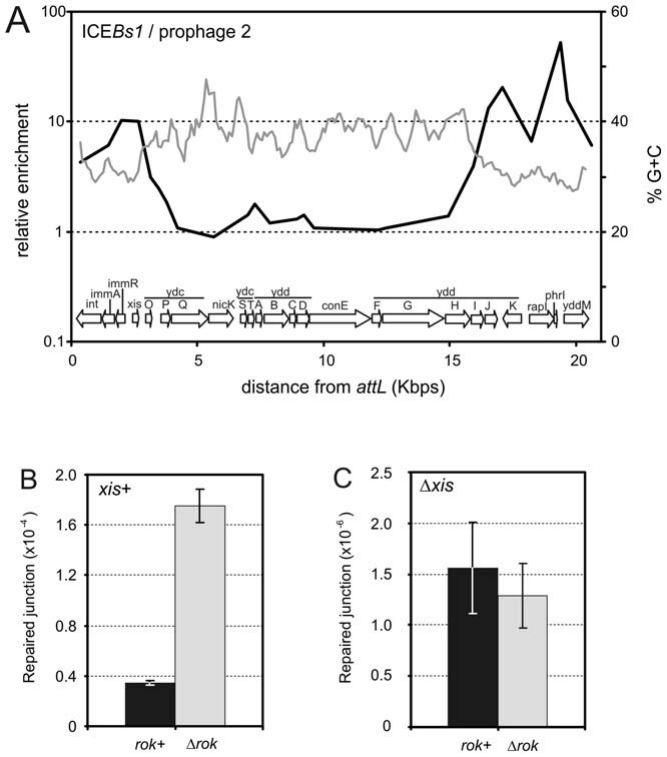
Association of Rok with ICE*Bs1.* A. Association of Rok-myc with ICE*Bs1* in relation to a sliding window analysis of its %G+C. The genomic organization of ICE*Bs1* is given in the graph. Data are from Figure 2. B. Excision of ICE*Bs1* in *rok* ^+^ (MMB869) and *rok^−^* (WKS1112) strains. C. Excision of an ICE*Bs1 xis* null mutant in *rok* ^+^ (CAL873) and *rok* ^−^ (WKS1093) strains. Strains used in panels B and C contained an (uninduced) *amyE*::*Pxyl-rapI* construct. Induction of this construct eliminated the effect of a *rok* mutation (data not shown). For panels B and C, error bars indicate the standard error of the mean (n = 3).

Rok binds to regions of the mobile element ICE*Bs1*, and the regions with the most binding, at the left and right ends of ICE*Bs1*, have the lowest G+C content (Figure 5A). The genes encoding the ICE*Bs1* site-specific recombinase (*int*) and the excisionase (*xis*) that allow excision from the genome are in the left end, and the recombination reaction occurs between 17 bp sequences found at the ends of the integrated element (*attL* and *attR*) [12]. During normal exponential growth, ICE*Bs1* gene expression is repressed by the element's repressor ImmR and there is very little excision of ICE*Bs1* [31]. Upon production of active RapI, a cell sensory protein, or during the RecA-dependent SOS response, ImmR is inactivated and ICE*Bs1* gene expression is derepressed. This leads to rapid production of excisionase and efficient excision of ICE*Bs1* from the chromosome [31], [32]. Because Rok was associated with genes and sequences at the ends of ICE*Bs1*, we determined the effects of a *rok* null mutation on the stability of ICE*Bs1*.

We found that endogenous excision of ICE*Bs1* increased approximately 4-fold in a *rok* null mutant (Figure 5B). Excision of ICE*Bs1* by site-specific recombination between the left and right attachment sites (*attL* and *attR*) leaves behind an empty bacterial attachment site, *attB*, that is readily detected by PCR using appropriate primers [12]. The occurrence of *attB* in a population, compared to a nearby chromosomal locus *ydbT*, is a measure of the frequency of excision of ICE*Bs1* [33], [34]. During exponential growth, the excision frequency of ICE*Bs1* in *rok* ^+^ cells was ∼4×10^−5^ (Figure 4B), consistent with previous measurements [33], [34]. In a *rok* null mutant, this frequency increased to ∼1.6×10^−4^ (Figure 5B).

The increased excision frequency of ICE*Bs1* in the *rok* null mutant could be due to an increase in integrase- and excisionase-mediated site-specific recombination, or possibly homologous recombination between the 60 bp direct repeats that mark the ends of ICE*Bs1*. We found that the increased excision in the *rok* null mutant was dependent on the ICE*Bs1* excisionase. An ICE*Bs1 xis* (excisionase) null mutation reduced the excision frequency to ∼1-2×10^−6^ (Figure 5C), consistent with previous findings [33]. This frequency did not significantly change in a *rok* null mutant (Figure 5C), indicating that the increased spontaneous excision observed in the *rok* mutant requires excisionase and is not due to increased homologous recombination between the 60 bp direct repeats at the ends of ICE*Bs1*. Based on these results, we conclude that Rok contributes to keeping ICE*Bs1* quiescent in the genome.

## Discussion

Results presented here show that *B. subtilis* Rok binds A+T-rich DNA, that the DNA binding activity resides in its C-terminal region, and that Rok helps inhibit the mobile genetic element ICE*Bs1*. Together with previous work on the effects of *rok* on gene expression [8], these properties indicate that Rok is analogous to the nucleoid-associated protein H-NS from *E. coli* and other Gram negative organisms [3]–[5], and the recently described Lsr2 of Mycobacterium and related high-G+C actinomycetes [6].

### Targets of Rok and horizontal gene transfer

Rok is a negative regulator of genes involved in horizontal gene transfer in at least two ways: 1) as a repressor of competence development, and 2) as an inhibitor of expression of genes in A+T-rich chromosomal regions. *rok* was initially identified as a repressor of of *comK* [9]. The *comK* gene product is a transcriptional activator and the master regulator of competence development and the K-state in *B. subtilis* [10], [11], [35]. By virtue of repressing *comK*, Rok also indirectly represses expression of the genes activated by ComK and is a therefore a strong negative regulator of competence development, thus inhibiting the ability of cells to acquire foreign DNA.

Rok also binds to chromosomal regions that are known or thought to have been acquired by horizontal gene transfer and are characterized by a high A+T content. One of these regions contains ICE*Bs1*, an integrative and conjugative element integrated in the *B. subtlis* chromosome [12]. Rok binds to ICE*Bs1* and helps prevent spontaneous excision. Many other horizontally acquired genes are bound by Rok in vivo and expression of some of these were previously found to be de-repressed in a *rok* null mutant [8]. Interestingly, *rok* itself appears to have been recently acquired in the *B. subtilis - B. amyloliquefaciens - B. licheniformis* clade as it is inserted in and interrupts an otherwise conserved genomic arrangement [8]. *rok* is auto-regulated, repressing its own expression. That is, transcription of *rok* increases in the absence of functional Rok protein. Thus, when the concentration of Rok decreases at its own promoter, its expression will increase, perhaps allowing cells to adjust the levels of Rok in response to acquisition of new DNA that has a high A+T content.

### Effects of Rok on gene expression

A null mutation in *rok* causes increased expression of at least 20 transcription units, either directly or indirectly [8], separate from genes that are activated by ComK. In vitro, Rok binds to sequences upstream of at least a subset of these genes [8]. We found that in vivo Rok is associated with most of these genes, consistent with the notion that Rok directly represses their transcription.

Our results indicate that the number of chromosomal genes bound by Rok is s significantly greater than the number of genes whose expression is detectably altered in a *rok* null mutant [8]. There are two ways to explain this difference. First, many genes bound by Rok are not expected to be expressed under the conditions used for analysis of mRNA levels [8]. This is particularly true for genes that might be controlled by other regulators, and for regions containing the mobile genetic elements that are strongly repressed during normal growth. Second, Rok may be bound to regions but not properly positioned to have an effect on transcription.

### Multiple ways for Rok to bind DNA?

There is a general correlation between chromosomal regions with low G+C content and Rok binding. However, it is notable that at least a few of the chromosomal regions bound by Rok appear to have a G+C content much closer to or greater than the norm, including the *comK* and *rok* regulatory regions. Rok might be recruited to these regions by other DNA binding proteins. Alternatively, Rok might be capable of some sequence-specific binding somewhat different from binding to A+T-rich DNA, and these possibilities are not mutually exclusive. A discriminative MEME motif search [36] using the few regions of Rok binding that are close to the average G+C content identified a motif that appears to be overrepresented (Figure S2). This motif could be a binding site for another protein that possibly interacts with Rok, or could indicate a specific binding sequence for Rok. Further genetic and functional dissection of Rok and this motif should help determine how Rok is associated with this DNA. Binding of H-NS to DNA also appears to be complex. H-NS can interact with or bind to regions bound by other DNA binding proteins, might have some site-specific binding, and can switch from stimulating DNA bridging to causing DNA stiffening {e.g., [5], [37]–[40]}.

### Rok and the nucleoid-associated proteins H-NS and Lsr2

All bacteria appear to have nucleoid-associated proteins (NAPs) [41] that are abundant, bind relatively non-specifically to extended regions of the chromosome, and often cause changes in DNA topology. The most highly conserved nucleoid-associated protein is the “heat-unstable” protein HU (and the related integration host factor IHF) found in both Gram negative and Gram positive organisms. In contrast, the heat-stable nucleoid restructuring protein H-NS is found in several Gram negative bacteria, but there are no obvious homologues in Gram positive bacteria (reviewed in [42]).

Despite the lack of sequence similarity, the recently characterized Lsr2 protein from *Mycobacerium* and related high G+C Gram positive bacteria is a functional analogue of H-NS [6]. Like H-NS, Lsr2 binds A+T-rich DNA, including regions acquired by horizontal gene transfer [7], [43], can repress transcription [43], is capable of bridging DNA [6], [44], and can partly substitute for H-NS in *E. coli* [6]. Neither H-NS nor Lsr2 homologues have been found in low G+C content Gram positive species.

There are several functional similarities between Rok of *B. subtilis* and H-NS (and Lsr2). Both Rok and H-NS act as negative regulators of transcription and bind extended chromosomal regions with high A+T content. H-NS causes significant changes in DNA topology, and this has also been postulated for Rok [27]. Most notably, both Rok and H-NS help silence foreign DNA with a high A+T content. Transcriptional repression exerted by H-NS can be reversed by certain anti-silencing mechanisms {reviewed in [45]}. Likewise, auto-activation of *comK* transcription is accomplished by preventing Rok-mediated repression [27]. Rok and H-NS are both relatively small (20.7 kDa and 15.4 kDa, respectively), although H-NS appears to be more abundant than Rok. There are approximately 20,000 molecules per cell of H-NS in growing cells [4]. In contrast, we estimate that there are approximately 1,000–3,000 molecules of Rok per genome of exponentially growing *B. subtilis* cells (see Materials and Methods). Based on the several similarities in function, we propose that Rok of *B. subtilis* and its close relatives is functionally analogous to H-NS of Gram negative bacteria and Lsr2 of Mycobacterium and related high G+C actinomycetes. We suspect that other organisms have H-NS analogues that are not readily recognized by sequence similarities.

## Materials and Methods

### Media and growth conditions

For routine growth and manipulations, *E. coli* and *B. subtilis* cells were grown in LB medium. For most experiments, *B. subtilis* cells were grown in the MOPS buffered S7_50_ defined minimal medium [46] with 0.1% glutamate, supplemented with required amino acids (typically tryptophan and phenylalanine), 1% glucose or arabinose as a carbon source, and 1 mM IPTG or 1% xylose as inducer as necessary. Strains with plasmids integrated into the chromosome by single crossover were grown with appropriate antibiotic to maintain selection for the integrated plasmid.

### Strains and alleles

*B. subtilis* strains used are listed in Table 3. PCR Primers used in strain constructions are listed in Table S1. *B. subtilis* strains were constructed by transformation using standard procedures [35], [47] Previously described alleles affecting ICE*Bs1* include *Δxis190*, a deletion of the excisionase gene of ICE*Bs1* [33], and Pxyl-*rapI* [48], used to induce efficient ICE*Bs1* gene expression.

**Table 3:** *B. subtilis* strains.

Strain	Relevant genotype
168	*trpC2*
JH642	*trpC2 pheA1*
AG2030	168 *rok*::pIYFP-rok (*cat*) (*rok-iyfp*)
CAL873	JH642 Δ*xis190* Δ(*rapI-phrI*)*342*::*kan amyE*::*Pxyl-rapI*
MMB869	JH642 *amyE*::*Pxyl-rapI*
WKS1030	JH642 Δ*rok*::*cat*
WKS1093	JH642 Δ*xis190* Δ(*rapI-phrI*)*342*::*kan amyE*::*Pxyl-rapI rok*::*cat*
WKS1102	168 *rok*::pIYFP-rok (*cat*::*ery*) *hbs*::pWKS934 (*cat*)
WKS1112	JH642 *amyE*::*Pxyl-rapI rok*::*cat*
WKS703	168 *rok*::pIYFP-rok (*cat*::*ery*) (*rok-iyfp*)
WKS895	168 *rok*::pWKS516 (*spc*) (*rok-myc*)
WKS938	168 *hbs*::pWKS913 (*spc*) (*hbs-myc*)
WKS942	168 *hbs*::pWKS934 (*cat*) (*hbs-mCherry*)

#### *rok-iyfp*

The 3′ end of the *rok* open reading frame was amplified using primers rok-gfp-KpnI and rok-gfp -EcoRI. After digestion with KpnI and EcoRI, this fragment was ligated into similarly digested pIYFP [14], yielding pIYFP-Rok. To allow for dual labeling, the chloramphenicol resistance gene of AG2030 (*rok-yfp*) was replaced with a erythromycin resistance gene using plasmid pCm::Em [49].

#### *hbs*-*mCherry*

The 3′ end of the *hbs* open reading frame was amplified using primers oWKS-213 and oWKS-215. After digestion with EcoRI and XhoI, the fragments were ligated into similarly digested pWKS553, yielding pWKS934. pWKS553 is derived from pGEMcat [50] and contains *mCherry* inserted between the KpnI and SphI sites. *mCherry* was amplified with primers oWKS-114 and oWKS-115 from pMMB1010 (from M. Berkman, Suffolk University, Boston).

#### *rok-myc* and *hbs-myc*

The 3′ end of *rok* and *hbs* were amplified using primers oWKS-121 or oWKS-213 and oWKS-215, respectively. After digestion with EcoRI and XhoI, the fragments were ligated into similarly digested pCAL812 (a vector for making C-terminal fusions to 3x-myc and encoding spectinomycin-resistance for selection of single cross-over integrants in *B. subtilis*), yielding pWKS516 and pWKS913, respectively. These plasmids were introduced by single crossover into the *B. subtilis* chromosome by natural transformation selecting for spectinomycin-resistance.

#### *Δrok::cat*

We used long flanking homology PCR [51] to construct a deletion-insertion that replaces the *rok* open reading frame with a chloramphenicol resistance cassette. Fragments ∼1 kb upstream and downstream of *rok* were amplified using primers oWKS-245, oWKS-246, and oWKS-247 and oWKS-248, respectively, resulting in two products with regions of complementarity to a chloramphenicol resistance cassette (*cat*). These products were used as megaprimers in an expand Long Template PCR reaction (Roche) with plasmid pGEMcat (Youngman, 1989) as template, followed by a second Expand Long Template PCR using primers oWKS-245 and oWKS-248. Purified PCR product of the expected size was transformed into a wild type strain, and double crossover replacement of the *rok* gene with the *cat* cassette was verified by PCR.

#### *rok-myc* alleles in *E. coli*

*E. coli* strains expressing C-terminally myc-tagged variants of Rok were constructed as follows. Region I (N-terminal) was amplified using primers oWKS-235 and oWKS-239, region II (central) using primers oWKS-236 and oWKS-240, region III (C-terminal) using primers oWKS-237 and 238, region I-II using primers oWKS-235 and oWKS-240, region II-III using primers oWKS-236 and oWKS-238, and full length *rok* (region I-II-III) was amplified using primers oWKS-235 and oWKS-238. The resulting PCR products (that contain an artificial ribosome binding site and a sequence encoding a C-terminal cMyc epitope tag) were digested with SalI and SphI and cloned into similarly digested pDR110, yielding pWKS1046 (region I), pWKS1048 (region II), pWKS1049 (region III), pWKS1051 (region I+II), pWKS1052 (region II+III) and pWKS1054 (full length). After initial transformation into an intermediary *E. coli* host, the plasmids were recovered and introduced into *E. coli* BL21(DE3) [52] for the ChIP experiments.

### Fluorescence microscopy

Cells were grown in defined minimal medium, placed on agarose (1.5%) pads containing Spizizen minimal salts [47]. DAPI was added to a final concentration of ∼80 ng/ml five minutes prior to visualization. Images were acquired using Nikon Ti-E inverted microscope under a 100× phase oil objective. Fluorescence images were acquired using Nikon Intensilight mercury illuminator and appropriate sets of excitation and emission filters (49008 for mCherry, 49003 for YFP and 49000 for DAPI, Chroma). Images were recorded using a CoolSNAP HQ camera (Photometrics) and processed using NIS-Elements Advanced Research 3.10 Software. TIFF images were processed in Adobe Photoshop CS3 and Figure 1 was prepared in Adobe Illustrator CS3.

### Chromatin immunoprecipitation (ChIP) experiments

Chromatin immunoprecipitation of DNA bound to the various proteins in *B. subtilis* was done essentially as described [53], except that DNA was precipitated in the presence of glycogen (20 µg) as a carrier. For ChIP experiments in *E. coli*, crosslinking was done at room temperature for 20 minutes. Myc-tagged proteins were immunoprecipitated using monoclonal anti-cMyc antibodies (Zymed). Both Rok-myc and HBsu-myc were functional in *B. subtilis* (see results) and both were detected in Western blots (data not shown). Preliminary comparisons between Rok-myc and HBsu-myc in Western blots with anti-myc monoclonal antibodies indicated that there is about 20-50-fold more HBsu in the cell than Rok. Since the cellular concentration of HBsu is about 50,000 molecules per genome [54], we estimate that there are approximately 1,000–3,000 molecules of Rok per genome. For Rok-myc and HBsu-myc, we verified that the anti-myc antibodies actually immunoprecipitated the protein by depleting it from an extract (data not shown). Even though there was little or no significant enrichment of specific chromosomal regions in the HBsu-myc ChIP experiments, the protein appeared to crosslink to DNA as the signals on the microarrays were significantly above background.

ChIP-chip analyses were performed as described previously using printed DNA microarrays with PCR products corresponding to most open reading frames and many intergenic regions [55]. The microarray data discussed in this publication have been deposited in NCBI's Gene Expression Omnibus [56] and are accessible through GEO Series accession number GSE23199 (http://www.ncbi.nlm.nih.gov/geo/query/acc.cgi?acc=GSE23199).

qPCR was performed on a Roche LightCycler 480 II. Samples (2 µl) of immunoprecipitated DNA were analyzed in duplicate in a 20 µl reaction volume that contained Sybr green. Signals were analyzed using the LightCycler 480 SW 1.5 software (Roche), according to the manufacturer (Absolute quantification; 2^nd^ derivative of Max). Signals were normalized against a 12-point standard curve obtained from a dilution series of total chromosomal DNA of *B. subtilis* JMA222 [34] or BL21(DE3) [52]. Primers used for real time PCRs are listed in Table S1.

For all conditions at least three independent biological replicates were analyzed and data shown represent the average of these replicates.

### ICE*Bs1* excision assay

Excision of ICE*Bs1* was monitored by quantitative real time PCR, as described previously [34] using primers CLO261, CLO262, CLO283, and CLO284 (Table S1).

### In silico analyses

Conserved regions of Rok from *B. subtilis* 168 (NP_389307.1), *B. amyloliquefaciens* FZB42 (YP_001420994.1), *B. pumilus* ATCC7061 (ZP_03052836.1) and SAFR-032 (YP_001486564.1), *B. licheniformis* ATCC14580 (YP_078814.1 and YP_079175.1), *B. psuedomycoides* DSM12442 (ZP_04153718.1) and *B. coagulans* 36D1 (ZP_04433348.1) were identified using ClustalW2 (http://www.ebi.ac.uk/Tools/clustalw2/index.html). Amino acids 96–191 of *B. subtilis* Rok were used to query the I-Tasser server (http://zhanglab.ccmb.med.umich.edu/I-TASSER) [57] and the PHYRE Protein Fold Recognition Server (http://www.sbg.bio.ic.ac.uk/~phyre/) [29] for secondary structure predictions. Sliding window analyses were performed using SWAAP 1.0.3 (http://asiago.stanford.edu/SWAAP/SwaapPage.htm) [58] on genome sequences of *Bacillus subtilis* (accession number AL009126) and *E. coli* BL21(DE3) (accession number CP001665) retrieved from GenBank (ftp://ftp.ncbi.nih.gov/genbank/genomes/Bacteria/). A discriminative MEME motif search [36] was used to detect overrepresented motifs in selected regions that show Rok binding in vivo.

## Supporting Information

Figure S1 Sequence alignment of Rok homologs. Amino acid sequences of Rok homologs from *B. subtilis* 168 (NP_389307.1), *B. amyloliquefaciens* FZB42 (YP_001420994.1), *B. pumilus* ATCC7061 (ZP_03052836.1) and SAFR-032 (YP_001486564.1), *B. licheniformis* ATCC14580 (YP_078814.1 and YP_079175.1), *B. psuedomycoides* DSM12442 (ZP_04153718.1) and *B. coagulans* 36D1 (ZP_04433348.1) were aligned using ClustalW2 (http://www.ebi.ac.uk/clustalw/). A. Dendrogram based on the ClustalW2 alignment. B–D. JalView visualization of the different Rok regions based on the ClustalW2 alignment. B. Region I. C. Region II. D. Region III.
(1.63 MB TIF)

Figure S2 Sequence Logos of a direct repeat enriched in selected sequences that bind Rok. Sequence logos are derived from a discriminative MEME search (http://meme.nbcr.net) using the *comK* and *rok* regulatory regions, the intergenic region between *ydeO* and *ydzF*, and the regulatory region of *sboA* as positive, and the regulatory regions of *comG* and *skfA* as well as an internal fragment of the *rok* gene as negative control sequences. A–B. Two highly similar 6-bp motifs identified by MEME. C. Sequences with a direct repeat (boxed) of motifs similar to those in panels A and B. On top of the sequences a sequencelogo of the entire motif is presented. P-values are calculated by MEME.
(7.22 MB TIF)

Table S1 Primers used.
(0.07 MB DOC)
