# Supplementary figures and images for: The Transcriptional Regulator Rok Binds A+T-Rich DNA and Is Involved in Repression of a Mobile Genetic Element in Bacillus subtilis
Source: PLoS Genet. 2010 Nov 11;6(11):e1001207. doi: 10.1371/journal.pgen.1001207 (PMC2978689; doi:10.1371/journal.pgen.1001207)

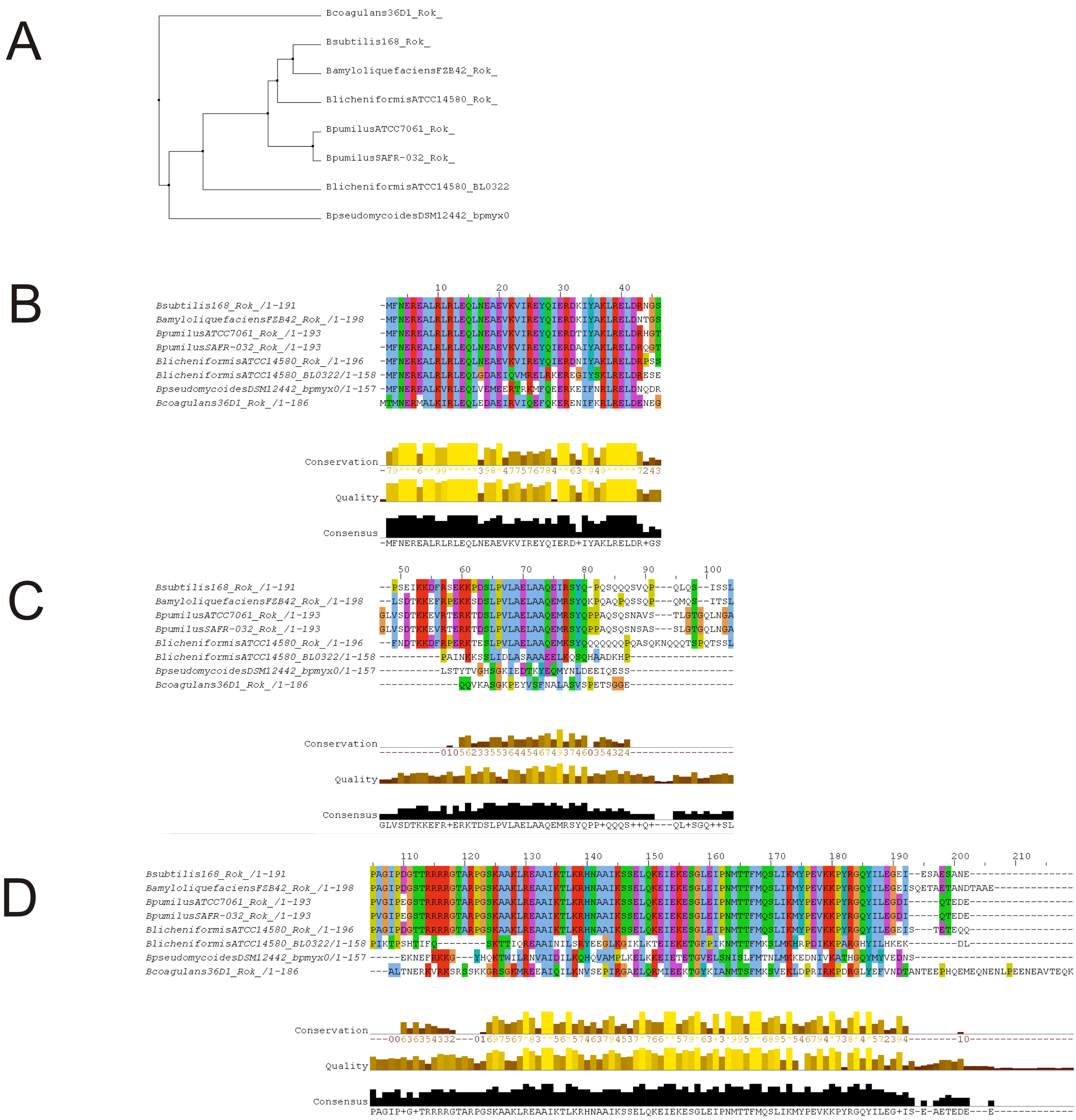

Supplement: Figure S1 — Sequence alignment of Rok homologs. Amino acid sequences of Rok homologs from B. subtilis 168 (NP_389307.1), B. amyloliquefaciens FZB42 (YP_001420994.1), B. pumilus ATCC7061 (ZP_03052836.1) and SAFR-032 (YP_001486564.1), B. licheniformis ATCC14580 (YP_078814.1 and YP_079175.1), B. psuedomycoides DSM12442 (ZP_04153718.1) and B. coagulans 36D1 (ZP_04433348.1) were aligned using ClustalW2 (http://www.ebi.ac.uk/clustalw/). A. Dendrogram based on the ClustalW2 alignment. B–D. JalView visualization of the different Rok regions based on the ClustalW2 alignment. B. Region I. C. Region II. D. Region III. (1.63 MB TIF) [file pgen.1001207.s001.tif]

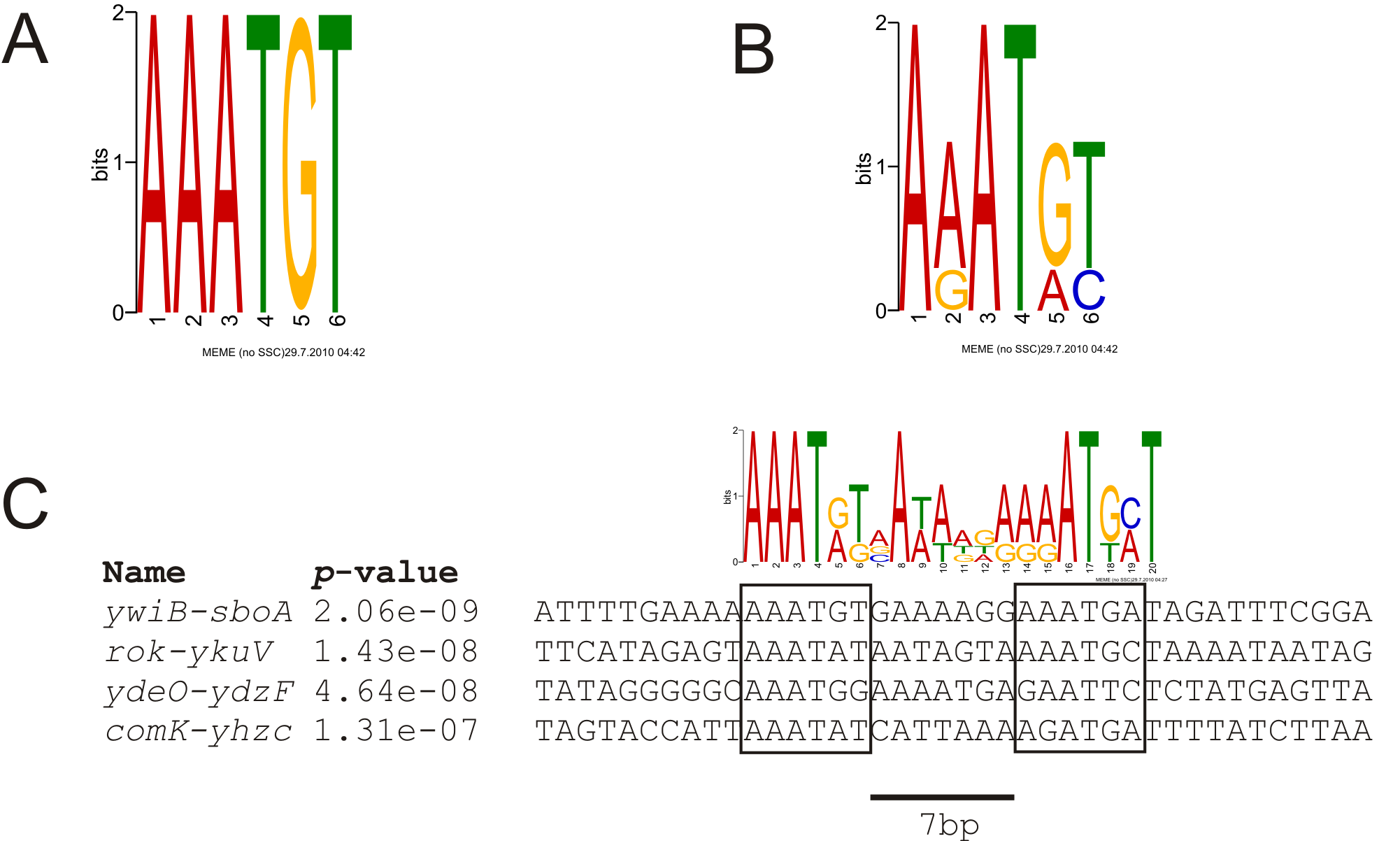

Supplement: Figure S2 — Sequence Logos of a direct repeat enriched in selected sequences that bind Rok. Sequence logos are derived from a discriminative MEME search (http://meme.nbcr.net) using the comK and rok regulatory regions, the intergenic region between ydeO and ydzF, and the regulatory region of sboA as positive, and the regulatory regions of comG and skfA as well as an internal fragment of the rok gene as negative control sequences. A–B. Two highly similar 6-bp motifs identified by MEME. C. Sequences with a direct repeat (boxed) of motifs similar to those in panels A and B. On top of the sequences a sequencelogo of the entire motif is presented. P-values are calculated by MEME. (7.22 MB TIF) [file pgen.1001207.s002.tif]
