# Supplementary material for: The Transcriptional Regulator Rok Binds A+T-Rich DNA and Is Involved in Repression of a Mobile Genetic Element in Bacillus subtilis
Source: PLoS Genet. 2010 Nov 11;6(11):e1001207. doi: 10.1371/journal.pgen.1001207 (PMC2978689; doi:10.1371/journal.pgen.1001207)
Supplement: Table S1 — Primers used. (0.07 MB DOC) [file pgen.1001207.s003.doc]

**Table S1. Primers used.**

| **Primer name** | **Sequence (5' - 3')** | **Information/use** |
| --- | --- | --- |
| CLO261 | GCCTACTAAACCAGCACAAC | attB qPCR |
| CLO262 | AGCAAGTCTTCTCCCATAGC | attB qPCR |
| CLO284 | CTTCCGCACATGCTCCGAAC | ydbT qPCR |
| CLO285 | TCGGCAGCAGGATCACTGAC | ydbT qPCR |
| oWKS-114 | GGGGTACCCCGGATCCCTCGAGGGAGAAGGACAAGGACAAGGACAAGGACCAGGACGAGGATACGCATACCGAAGCGGTTCCGGAATGGTTTCCAAGGGCGAGGAGGA | amplify mCherry |
| oWKS-115 | ACATGCATGCATGTTTATTATTTGTACAGCTCATCCAT | amplify mCherry |
| oWKS-120 | GGAATTCAGAGAGCTGGACAGAAATGG | amplify 3’ fragment of rok |
| oWKS-121 | CCGCTCGAGTTCGTTTGCTGATTCTG | amplify 3’ fragment of rok |
| oWKS-213 | GGAATTCAATGCGGTTGCAGAAGCAAG | amplify 3’ fragment of hbs |
| oWKS-215 | CCGCTCGAGTCGAGTTTTTCGGAATGGTG | amplify 3’ fragment of hbs |
| oWKS-235 | ACGCGTCGACATTAAAGAGGAGAAATTAACCATGTTTAATGAAAGAGAAGCTTTG | amplify part of rok from region I with artificial RBS |
| oWKS-236 | ACGCGTCGACATTAAAGAGGAGAAATTAACCATGCCTTCCGAAATCAAAAAGGATTTC | amplify part of rok from region II with artificial RBS |
| oWKS-237 | ACGCGTCGACATTAAAGAGGAGAAATTAACCATGCCTGCCGGTATACCAGACGGAACA | amplify part of rok from region III with artificial RBS |
| oWKS-238 | ACATGCATGCTTATTAGCTACTATTAAGATCCTC | amplify part of rok including region III with C-terminal myc-tag |
| oWKS-239 | ACATGCATGCTTATTAGCTACTATTAAGATCCTCCTCGGATATTAACTTCTGCTCACCGTTGAGGTCTTCCTCACTGATCAATTTCTGTTCTCCATTCAAGTCCTCTTCAGAAATGAGCTTTTGCTCAGAGCGGCCCTCGAGGCTTCCATTTCTGTCCAGC | amplify part of rok including region I with C-terminal myc-tag |
| oWKS-240 | ACATGCATGCTTATTAGCTACTATTAAGATCCTCCTCGGATATTAACTTCTGCTCACCGTTGAGGTCTTCCTCACTGATCAATTTCTGTTCTCCATTCAAGTCCTCTTCAGAAATGAGCTTTTGCTCAGAGCGGCCCTCGAGAAGAGAGGAAATAGATTGAAGCTG | amplify part of rok including region II with C-terminal myc-tag |
| oWKS-245 | AGCCCATGCCGGAATTG | amplify fragment upstream of rok |
| oWKS-246 | TACCGCACAGATGCGTAAGGAGTCCTCAATGTACCCCCTATC | amplify fragment upstream of rok, for inserting cat |
| oWKS-247 | TAATATGAGATAATGCCGACTGTACTAAATATAAAGAAAAACTGCTTGGCATC | amplify fragment downstream of rok, for inserting cat |
| oWKS-248 | AACTCCAGCAGCTGTC | amplify fragment downstream of rok |
| oWKS-249 | CTGTCCACAGGAGTCTATTG | manC qPCR |
| oWKS-250 | GAAGACCATGCTCCTACATC | manC qPCR |
| oWKS-251 | GCTGGCTGACCTTGTCTATC | rhsE qPCR |
| oWKS-252 | TTGGCGGAGCTCATAATGGG | rhsE qPCR |
| oWKS-253 | CCGCTGGAGTTAGAATCTAC | ybcL qPCR |
| oWKS-254 | TGCTGTTCAAGGCCGAAATG | ybcL qPCR |
| oWKS-255 | GATAAGCGAGTCGACATCTG | yncH/vgr qPCR |
| oWKS-256 | AGACACCCGTTTCTCTGAAG | yncH/vgr qPCR |
| oWKS-257 | CGCCTTACCCTTAACCAGTC | ybbA qPCR |
| oWKS-258 | GTTGCAGGCGAAAGTGTTTG | ybbA qPCR |
| oWKS-259 | CCCGTCTGTTTACAGGTTTC | phnO qPCR |
| oWKS-260 | GGCAATCCTGTGGAGTATAG | phnO qPCR |
| Rok-gfp-KpnI | GGGGTACCCTAGAAAGCCCCTTAATCCC | upstream primer to fuse rok to iyfp |
| Rok-gfp-EcoRI | GGAATTCACCCTCTAAAATGTACTGC | downstream primer to fuse rok to iyfp |
